# Risk Factors for Rapid Recurrence of Hyperkalemia following Cessation of Sodium Zirconium Cyclosilicate

**DOI:** 10.3390/jcm11237096

**Published:** 2022-11-30

**Authors:** Teruhiko Imamura, Nikhil Narang, Koichiro Kinugawa

**Affiliations:** 1 Second Department of Medicine, University of Toyama, Toyama 930-0194, Japan; 2 Advocate Christ Medical Center, Oak Lawn, IL 60453, USA

**Keywords:** chronic kidney disease, potassium, hyperkalemia

## Abstract

**Background:** Sodium zircon`ium cyclosilicate (SZC), a recently introduced potassium binder, is indicated to treat hyperkalemia. SZC is often terminated soon after the normalization of hyperkalemia in real-world clinical practice. We aimed to investigate the risk factors for the rapid recurrence of hyperkalemia following cessation of SZC. **Methods:** Patients in whom SZC was discontinued following an improvement in hyperkalemia were followed up for three months. The baseline characteristics that were associated with the rapid recurrence of hyperkalemia (>5.0 mEq/L of serum potassium levels within 3 months) were investigated. **Results:** A total of 44 patients terminated SZC following the normalization of hyperkalemia. The median age was 81 (69, 87) years old, and 59% were men. When evaluating baseline characteristics, a higher dose of renin-angiotensin system inhibitors was significantly associated with the recurrence of hyperkalemia (adjusted hazard ratio of 1.26, 95% confidence interval 1.02–1.69, *p* = 0.045) at a designated cutoff of 2.5 mg/day of equivalent enalapril dose. **Conclusions:** SZC should be considered for ongoing continuation after normalization of hyperkalemia, particularly in patients receiving a relatively higher dose of renin-angiotensin system inhibitors.

## 1. Background

Sodium zirconium cyclosilicate (SZC) is a recently introduced non-polymer zirconium silicate agent, which decreases the levels of serum potassium by exchanging sodium and hydrogen for potassium and ammonium ions in the gastrointestinal tract with robust evidence [1].

In real-world practice, potassium-binding agents are often utilized transiently as a rescue therapy until improvement of hyperkalemia is attained [2]. Cessation of SZC may be associated with a higher risk of recurrent hyperkalemia [3]. An immediate recurrence of hyperkalemia should result in higher mortality and morbidity in patients [4].

Thus far, the factors associated with the recurrence of hyperkalemia following cessation of SZC have not been thoroughly investigated. It should be highly recommended to continue SZC therapy, particularly for those with some risk factors. In this study, we investigated the factors associated with the recurrence of hyperkalemia following cessation of SZC.

## 2. Methods

### 2.1. Patient Selection and Study Design

Patients who received SZC between July 2020 and October 2022 for hyperkalemia (defined as serum potassium level >5.0 mEq/L) were considered for inclusion in this retrospective study. Among them, patients in whom SZC was terminated following normalization of hyperkalemia were included. The day of SZC cessation was defined as day 0. Patients were followed up for 3 months from day 0. Patients who died or were lost to follow-up within the observational period were excluded. Informed consent was obtained from all participants, and the present study was approved by the Ethics Committee of the local institute.

### 2.2. SZC Therapy

Before day 0, SZC was administered to all patients. SZC was initiated to treat hyperkalemia at a loading dose for 2 days, in principle, followed by a maintenance dose of 5–15 g/day [5]. SZC was considered to be terminated following the amelioration of hyperkalemia at the discretion of the treating physicians.

### 2.3. Primary Outcome

The recurrence of hyperkalemia (defined as >5.0 mEq/L of serum potassium level) within 3 months following cessation of SZC was defined as a primary outcome. We investigated the risk factors for the recurrence of hyperkalemia through analysis of the baseline patient covariates.

### 2.4. Data Collection

Demographic, echocardiographic, laboratory and medication data retrieved just before the day of SZC cessation (day 0) were analyzed. Doses of medical therapy commonly observed in this patient sample were converted to equivalents of carvedilol, enalapril, spironolactone and furosemide [6].

### 2.5. Statistics

Statistics were conducted using SPSS Statistics 23 (SPSS Inc., Armonk, IL, USA). Two-tailed *p*-values < 0.05 were considered statistically significant. All variables were assumed as non-parametric data, considering the small sample size.

The trend in serum potassium levels following cessation of SZC was assessed by using the Friedman test. The Cox proportional hazard ratio regression analyses were performed to investigate the baseline characteristics that were associated with the recurrence of hyperkalemia within the 3-month observational period. Several potential risk factors for hyperkalemia were included in the univariable analyses. Variables with *p* < 0.05 in the univariable analyses were included in the multivariable analysis with a frothed method. Age and sex were included in the multivariable analysis, irrespective of their significance in the univariable analyses. A receiver-operating characteristics analysis was performed to calculate a cutoff of continuous variables for the estimation of the primary endpoint. Log-rank test was utilized to compare the cumulative incidence of recurrent hyperkalemia between the two groups.

## 3. Results

### 3.1. Baseline Characteristics

A total of 44 patients were analyzed in this analysis. The median age was 81 (69, 87) years old, and 59% were men (Table 1). All patients terminated SZC following the normalization of hyperkalemia. The potassium intake restriction was loosened for all patients following SZC cessation. No patients used sodium sparing salts. Twenty-seven patients (61%) had a history of chronic heart failure, and five patients (11%) were undergoing chronic hemodialysis. The median plasma B-type natriuretic peptide level was 154 (132, 205) pg/mL. Twenty-seven patients (61%) had been receiving renin-angiotensin system (RAS) inhibitors with a median dose of 1.25 (0, 3.75) mg/day enalapril equivalent. Ten patients (23%) had been receiving mineralocorticoid receptor antagonists.

### 3.2. The Trend in Serum Potassium Levels following SZC Cessation

**Figure 1:**
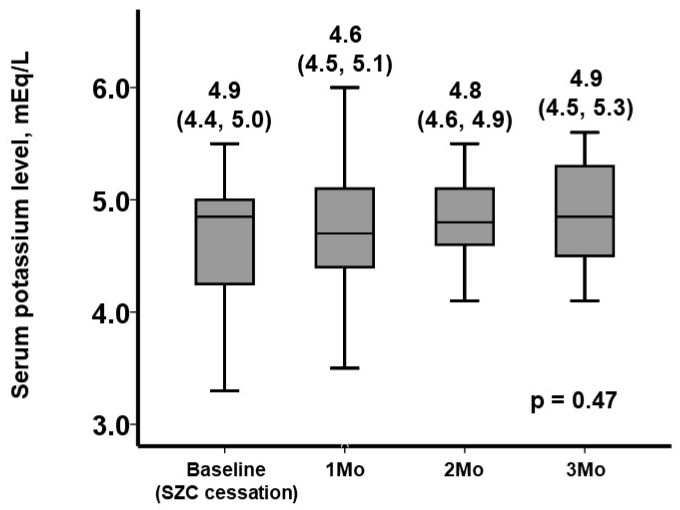
Three-month trends of serum potassium levels following SZC cessation.

Following the cessation of SZC, serum potassium levels increased overall during the 3-month observational period (*p* = 0.47; Figure 1). Among them, 24 patients experienced recurrent hyperkaliemia.

### 3.3. Rapid Recurrence of Hyperkalemia

Among the baseline covariates that were clinically suspected risk factors for recurrent hyperkalemia, plasma B-type natriuretic peptide (logarithmically transformed) and the dose of RAS inhibitors were significantly associated with the primary outcome in univariable analysis (*p* < 0.05 for both; Table 2). In the multivariable analysis, a higher dose of RAS inhibitors was associated with the primary endpoint (adjusted hazard ratio of 1.26 per 1.0 mg/day, 95% confidence interval 1.02–1.69, *p* = 0.045).

### 3.4. Stratification Using the Dose of RAS Inhibitors

A cutoff of the dose of RAS inhibitors for the primary endpoint was calculated as 2.5 mg/day, with area under the curve 0.81 (95% confidence interval 0.67–0.95, *p* = 0.001), sensitivity 0.67 and specificity 1.0.

**Figure 2:**
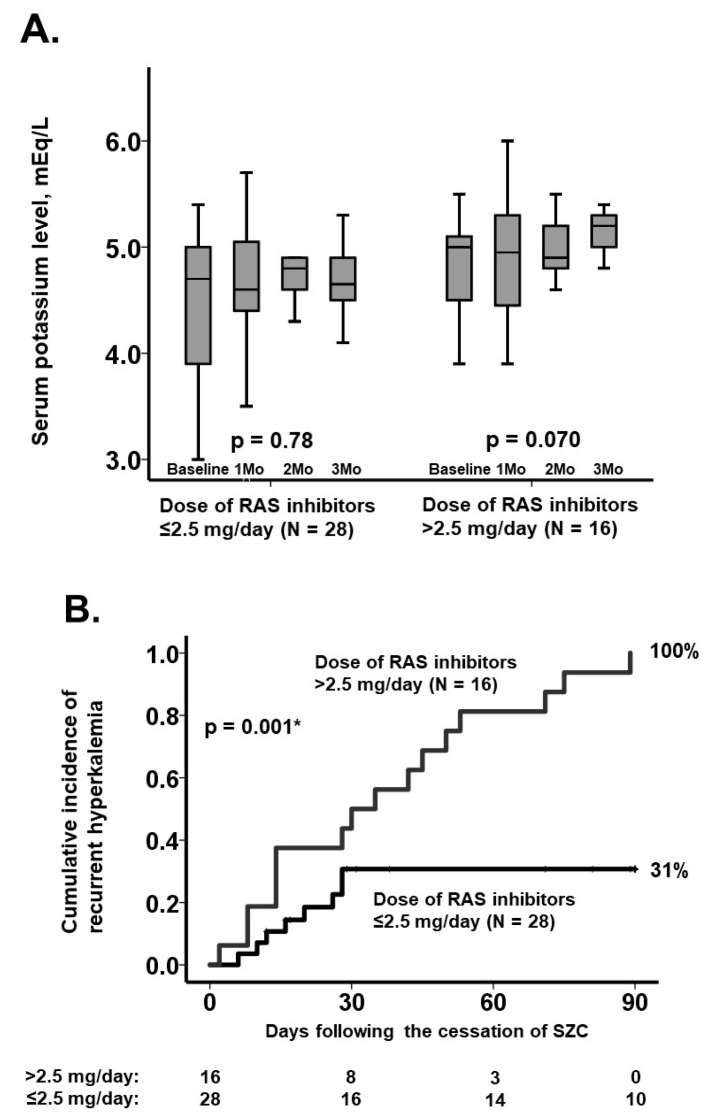
Three-month trends of serum potassium levels following SZC cessation (**A**) and cumulative incidence of recurrent hyperkalemia (**B**) stratified by the cutoff of baseline dose of RAS inhibitors. Trends were assessed by using the Friedman test, and cumulative incidences were compared by using the log-rank test. * *p* < 0.05.

Sixteen patients (36%) received RAS inhibitors >2.5 mg/day at baseline. Baseline eGFR were not significantly different between those with/without baseline RAS inhibitors >2.5 mg/day (*p* = 0.97). The median serum potassium level tended to increase following cessation of SZC among them (*p* = 0.070), whereas it remained unchanged among those with ≤2.5 mg/day of RAS inhibitors (*n* = 28, *p* = 0.78) (Figure 2A). Three-month cumulative incidence of recurrent hyperkalemia was higher in patients with >2.5 mg/day of RAS inhibitors compared with others (100% versus 31%, *p* = 0.001; Figure 2B).

**Figure 3:**
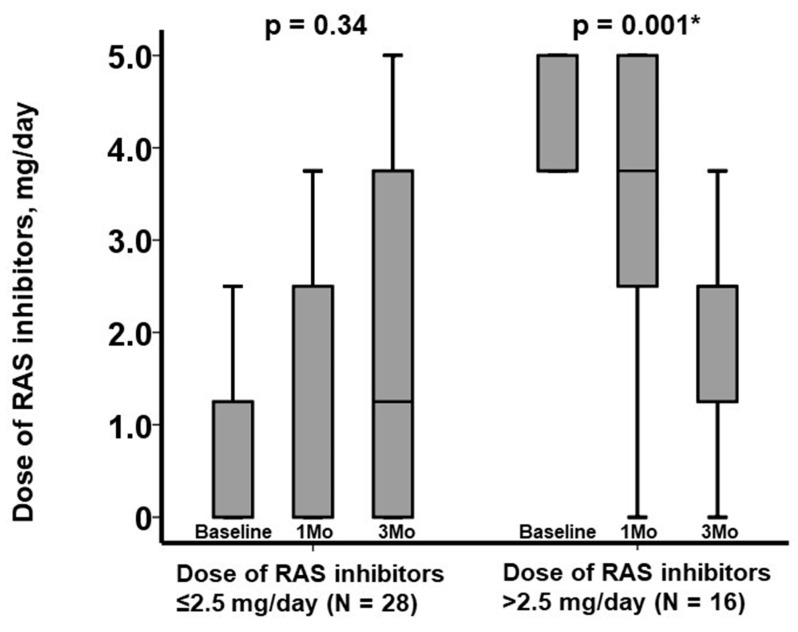
Three-month trends of dose of RAS inhibitors stratified by the cutoff of baseline dose of RAS inhibitors. Trends were assessed by using Friedman test. * *p* < 0.05.

The median dose of RAS inhibitors was observed to decrease significantly following cessation of SZC in patients with baseline dose >2.5 mg/day (*p* = 0.001), whereas it remained unchanged in patients with baseline dose ≤2.5 mg/day (*p* = 0.34) (Figure 3). The dose of loop diuretics remained unchanged following SZC cessation from 10 (0, 20) mg/day at baseline to 10 (0, 20) mg/day at 3-month follow-up (*p* = 0.67).

## 4. Discussion

### 4.1. Discussion of Our Findings

We demonstrated that a higher baseline dose of RAS inhibitors was a significant risk factor for the recurrence of hyperkalemia within 3 months following SZC cessation. We identified an enalapril equivalent dose of ≥2.5 mg/day as a cutoff point associated with recurrent hyperkalemia. We further observed that the down-titration of RAS inhibitors at this dose was commonly observed following SZC cessation.

Hyperkalemia is associated with mortality and morbidity in patients with a variety of comorbid conditions, including chronic heart failure, diabetes mellitus and chronic kidney disease [7]. In a large observational cohort of patients with chronic kidney disease and chronic heart failure who were on RAS inhibitors, 26% had hyperkalemia, and 18% had recurrent hyperkalemia within a 6-month follow-up, with a higher cumulative mortality risk in patients who had dose reductions or discontinuation of RAS inhibitors [8].

Potassium-binding agents are now available to manage hyperkalemia [9]. Of note, SZC has recently been introduced as a novel potassium-binding agent with robust evidence to rapidly normalize hyperkalemia and maintain serum potassium levels within the normal range with few drug-related adverse events [5].

Nevertheless, potassium-binding agents, including SZC, are often used as rescue agents in real-world practice, and they are terminated soon after the normalization of hyperkalemia in the majority of cases [2]. SZC cessation appears to be associated with recurrent hyperkalemia, which leads to downstream discontinuation or dose reduction in important therapies, which may ultimately worsen the outcomes in patients with chronic heart failure and kidney disease [3]. In this study, approximately half of the cohort encountered recurrent hyperkalemia within three months following SZC cessation.

Should SZC be continued in all patients with hyperkalemia despite the normalization of hyperkalemia? We observed in this study that a higher dose of RAS inhibitors was associated with the recurrence of hyperkalemia following SZC cessation. The dose of RAS inhibitors was decreased significantly following SZC cessation in patients with higher baseline doses of RAS inhibitors, likely due to an attempt to manage recurrent hyperkalemia without the use of SZC.

These therapies in patients with chronic heart failure and/or chronic kidney disease, however, significantly reduce the incidence of heart failure hospitalization and cardiovascular mortality [10]. We are now in a golden era of therapeutics for such cohorts where the combined administration of contemporary guideline-directed medical therapy, including angiotensin-neprilysin inhibitors, beta-blockers, mineralocorticoid antagonists and sodium-glucose co-transporter-2 inhibitors, significantly reduces the risk of heart failure hospitalizations and cardiovascular death [11]. Considering this as imperative to maximize these therapies in this patient population, given clear significant clinical benefits, therapies such as SZC should be incorporated in instances of medication-induced hyperkalemia to prevent dose reduction or discontinuation of these life-saving therapies.

### 4.2. Limitations

This is a proof-of-concept study, including a small sample size, which limits the power needed to observe significant associations of potential risk factors with recurrent hyperkalemia. Thus, we should emphasize that the dose of RAS inhibitors would not be the only risk factor for the recurrent hyperkalemia. Larger studies with wider variables might clarify more risk factors. Our results should be validated in larger scale multi-institutional studies. The timing of SZC cessation and dose adjustment of medical therapies were determined at the discretion of the physicians caring for the patient. Thus, the causality among factors, including SZC cessation, recurrence of hyperkalemia and dose adjustment of medical therapies, cannot be confirmed. Although this study aimed to assess the short-term incidence of recurrent hyperkalemia, data with longer follow-up are needed to better understand the prognostic impact of recurrent hyperkalemia. The serum potassium level might reach a new steady state at several months or years following SZC cessation, which remains the future concern to be studied.

### 4.3. Conclusions

A higher baseline dose of RAS inhibitors was associated with a recurrence of hyperkalemia within three months following SZC cessation. SZC therapy should be strongly considered for continuation following normalization of hyperkalemia to avoid discontinuation of disease-modifying therapies.

## Figures and Tables

**Table 1:** Baseline characteristics at the time of SZC cessation.

	*n* = 44
Demographics	
Age, years	81 (69, 87)
Men	26 (59%)
Body mass index	22.0 (20.2, 24.4)
Comorbidity	
Heart failure	27 (61%)
Diabetes mellitus	23 (52%)
Atrial fibrillation	11 (25%)
History of coronary intervention	18 (41%)
History of stroke	8 (18%)
Hemodialysis	5 (11%)
Laboratory	
Hemoglobin, g/dL	10.2 (9.0, 11.6)
Serum sodium, mEq/L	139 (137, 141)
Serum potassium, mEq/L	4.9 (4.3, 5.0)
Estimated glomerular filtration ratio, mL/min/1.73 m^2^	23.6 (18.3, 38.9)
Plasma B-type natriuretic peptide, pg/mL	154 (132, 205)
Echocardiography	
Left ventricular end-diastolic diameter, mm	49 (42, 54)
Left ventricular ejection fraction, %	49 (37, 65)
Left atrial diameter, mm	41 (34, 45)
Medication	
Beta-blocker	29 (66%)
Dose of beta-blocker, mg/day	2.5 (0, 10.0)
Renin-angiotensin system inhibitor	27 (61%)
Dose of renin-angiotensin system inhibitor, mg/day	1.25 (0, 3.75)
Mineralocorticoid receptor antagonist	10 (23%)
Dose of mineralocorticoid receptor antagonist, mg/day	0 (0, 0)
Loop diuretics	25 (57%)
Dose of diuretics, mg/day	10 (0, 20)

Continuous variables are stated as median and interquartile. Categorical variables are stated as numbers and percentage. Dose of beta-blocker is stated as carvedilol equivalent. Dose of renin-angiotensin system inhibitor is stated as enalapril equivalent. Dose of mineralocorticoid receptor antagonist is stated as spironolactone equivalent.

**Table 2:** Potential factors associated with recurrence of hyperkalemia within 3 months following SZC cessation.

	**Univariable Analysis**		**Multivariable Analysis**	
	**Hazard Ratio (95% CI)**	***p* Value**	**Hazard Ratio (95% CI)**	***p* Value**
Age, years	0.99 (0.96–1.02)	0.51	0.97 (0.94–1.05)	0.65
Male sex	1.67 (0.69–4.05)	0.25	1.76 (0.64–4.09)	0.34
Heart failure	0.81 (0.36–1.82)	0.61		
Hemodialysis	0.99 (0.30–3.34)	0.99		
eGFR, mL/min/1.73 m^2^	0.99 (0.98–1.02)	0.76		
Logarithm of plasma BNP, pg/mL	13.3 (1.70–103)	0.014 *	4.68 (0.46–40.5)	0.28
Left ventricular ejection fraction, %	1.01 (0.98–1.03)	0.72		
Dose of RAS inhibitor, mg/day	1.38 (1.11–1.72)	0.004 *	1.26 (1.02–1.69)	0.045 *
Dose of MRA, mg/day	1.03 (0.97–1.10)	0.36		
Dose of diuretics, mg/day	0.99 (0.97–1.03)	0.94		

CI, confidence interval; eGFR, estimated glomerular filtration ratio; BNP, B-type natriuretic peptide; RAS, renin-angiotensin system; MRA, mineralocorticoid receptor antagonist. * *p* < 0.05 by Cox proportional hazard ratio regression analysis.

## Data Availability

Data are available from the corresponding author upon reasonable requests.
